# Oral health and systemic inflammatory, cardiac and nitroxid biomarkers in hemodialysis patients

**DOI:** 10.4317/medoral.21629

**Published:** 2017-06-04

**Authors:** Jasna Cotič, Monika Ferran, Jasmina Karišik, Aleš Jerin, Pirkko J. Pussinen, Ana Nemec, Zlatko Pavlica, Jadranka Buturović-Ponikvar, Milan Petelin

**Affiliations:** 1 Department of Prosthodontics, Faculty of Medicine, University of Ljubljana, Ljubljana, Slovenia; 2 Department of Oral Medicine and Periodontology, Faculty of Medicine, University of Ljubljana, Ljubljana, Slovenia; 3 Institute of Clinical Chemistry and Biochemistry, University Medical Centre Ljubljana, Ljubljana, Slovenia; 4 Oral and Maxillofacial diseases, University of Helsinki, Helsinki, Finland; 5 Small Animal Clinic, Veterinary Faculty, University of Ljubljana, Ljubljana, Slovenia; 6 Department of Surgical and Radiological Sciences, School of Veterinary Medicine, University of California, Davis, USA; 7 Department of Nephrology, University Medical Centre Ljubljana, Ljubljana, Slovenia

## Abstract

**Background:**

Periodontal diseases have systemic inflammatory effects and have been adversely associated with cardiovascular diseases, which are also the most frequent cause of death in the end-stage renal disease. The aim of this cross-sectional study was to investigate the oral health and serum biomarkers among the hemodialysis (HD) patients in Slovenia.

**Material and Methods:**

111 HD patients were periodontally examined and their sera were assayed for C reactive protein (CRP), cardiac troponin T (TnT), nitrite/nitrate (NOx) and antibody levels to A. actinomycetemcomitans and P. gingivalis. The association of oral health with systemic response was analyzed with Kruskal-Wallis test, Fisher’s exact test and multivariate linear regression.

**Results:**

Bleeding on probing without periodontal pockets was present in 5.2%, calculus without periodontal pockets in 42.1%, shallow periodontal pockets in 39.5% and deep periodontal pockets in 13.2% of dentate patients. There were 28.8% edentulous participants. 63.1% of the patients had CRP levels higher than 3 mg/L and 34.2% higher than 10 mg/L. TnT was detectable in all participants, with 25.2% exhibiting levels higher than 100 ng/L. The median level of NOx was 43.1 µmol/L. Participants with higher CRP were more likely to be edentulous and have higher TnT levels. A direct association of oral health with TnT or NOx was not detected.

**Conclusions:**

HD patients in Slovenia have compromised oral health and increased serum inflammatory and cardiac biomarkers. Edentulousness was an independent predictor for the increased CRP, indicating a need for improved dental care to retain the teeth as long as possible.

** Key words:**Periodontal diseases, edentulousness, C reactive protein, cardiac troponin T, nitric oxide.

## Introduction

Periodontal diseases are infectious/inflammatory diseases affecting the supporting tissues of the teeth in susceptible individuals. Inefficient dental hygiene results in the formation of the microbial biofilm, causing gingival inflammation. In the advanced form, periodontal pockets between the tooth root and the adjacent gingiva and bone are formed, permanently flooding the bloodstream with bacteria, bacterial products such as lipopolysaccharide, and pro-inflammatory cytokines that can affect distant sites and organs (1). The most common pathogens associated with periodontitis are Gram-negative bacteria, principally *Aggregatibacter actinomycetemcomitans* (Aa) and *Porphyromonas gingivalis* (Pg). The association of periodontal diseases and systemic health is well documented, particularly the adverse effect on the cardiovascular health (2). Cardiovascular disease events are also the most common cause of death in the end-stage renal disease (ESRD) (3). In maintenance hemodialysis (HD) patients, periodontal diseases were associated with serum protein and electrolyte imbalance (4) and with systemic inflammation which might contribute to the cardiovascular disease-related death (5). In turn, kidney disease may also have detrimental effects on oral health as the patients are more susceptible to infections and more often experience gingivitis and periodontitis (6). An insight into their systemic health may be obtained by measuring changes in inflammatory and cardiovascular health biomarkers, among others C-reactive protein (CRP), cardiac troponin T (TnT) and nitric oxide (NO). CRP is an acute phase reactant, indicating distressed cells. Levels up to 3 mg/L are considered normal and levels over 10 mg/L appear in acute inflammation (7). Increased serum CRP also occurs in lower grade inflammatory processes, associating positively with the severity of periodontal diseases (8,9).

TnT is a cardiac biomarker indicating damage of the cardiac muscle and is a diagnostic tool in myocardial infarction. Modern high-sensitivity assays also detect it in many states without evident myocardial symptoms, possibly indicating subclinical myo-cardial necrosis or fibrosis. Elevated TnT is frequently encountered among patients with varying degrees of kidney disease (10) and has also been detected in patients with chronic periodontitis (11).

Periodontitis has also been associated with increased oxidative stress (12) and the production of NO (13). NO is both an important signal molecule as well as a cytotoxic effector molecule in nonspecific immune response (14). It regulates vascular tone through promoting smooth muscle relaxation and insufficient levels can contribute to high blood pressure, cardiac hypertrophy, and progression of kidney disease. But when produced in excess due to the general inflammatory state, reactive nitrogen species (RNS) can form, which are associated with atherosclerosis and hemodynamic instability (15). Even chronic swallowing of *P. gingivalis* alone has been shown to cause systemic inflammatory response by increased production of NO in mice (16).

ESRD is a complex condition and medically compromised patients are generally at greater risk for oral diseases which, in turn, may further jeopardize their health (17). The data on the extent and the underlying mechanisms of these interactions in HD patients is limited, especially concerning the levels of TnT and NO. The aim of this study was to investigate the status and relation-ships between the oral health and serum biomarkers among the HD patients in Slovenia.

## Material and Methods

This cross-sectional study recruited adult patients undergoing maintenance dialysis at the University Medical Centre Ljubljana, Slovenia, from January to November 2013. To be eligible for the study, a dialysis duration of at least 3 months was necessary. All the procedures were in accordance with the Helsinki Declaration of 1975, as revised in 1983. The study protocol was approved by the National Medical Ethics Committee of the Republic of Slovenia (No: 114/02/13). Informed consent was obtained from all individual participants.

-Questionnaire and oral health examination

Two dentists (MF and JK) visited the dialysis centre where they performed the interviews and periodontal examinations of the participants. The interview included a questionnaire with information on gender, age, general health status (history of diabetes mellitus, hypertension, regular intake of medications, smoking), number and frequency of visits to a dentist, and oral hygiene measures. Oral examination was carried out during the dialysis procedure under artificial light with the use of a plane mouth mirror and a WHO periodontal probe. The decayed-missing-filled index (DMF) was used to determine the number of decayed, missing and filled teeth. Wisdom teeth were also included. The amount of dental plaque and the gingival reaction to it were assessed with the approximal plaque index (API) and the sulcus bleeding index (SBI) by gently walking the periodontal probe through the gingival sulcus. Periodontal status was evaluated by the Community Periodontal Index of Treatment Need (CPITN). Each dental arch was divided into three sextants; probing was carried out around each tooth. Each sextant having at least two teeth not intended for extraction was scored from C0 to C4. If only one tooth remained in a sextant, it was added to the score of the nearby sextant. The highest score presented the overall score of a sextant. Community periodontal index (CPI) scores were used to determine the treatment need (TN) scores as shown in  Table 1.

**Table 1:**
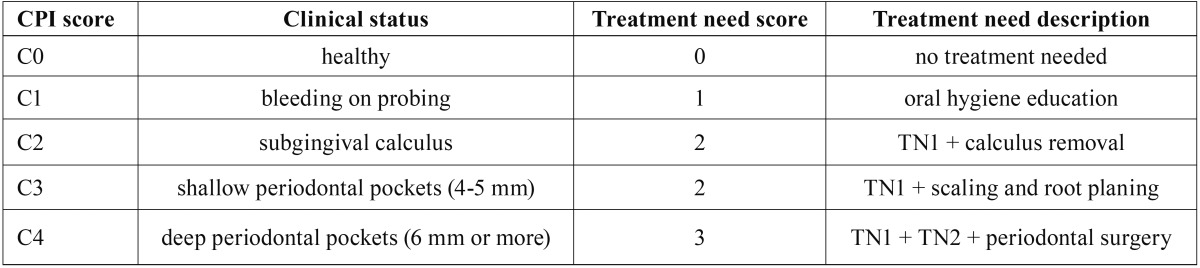
Community periodontal index of treatment need (CPITN) chart.

The kappa statistic was used to assess inter- and intra-examiner reproducibility. Dental examinations of 15 randomly selected individuals were carried out twice by both examiners. The second measurements were repeated after 2 weeks. Reproducibility of assessing DMF and all periodontal parameters was tested. Reliability was quantified by calculating the percentage of teeth, tooth sites or periodontal condition scores where both examiners agreed on. Intra-examiner calibration scores were 0.90 and 0.85, inter-examiner calibration score was 0.85.

-Serum biomarkers and antibody levels to *A. actinomycetemcomitans* and *P. gingivalis*

The serum from a pre-dialysis blood sample was separated by centrifugation (1,500 × g for 10 min) from the clotted blood and stored at −20°C. Concentration of CRP was measured using a chemiluminescent-immunometric high-sensitivity assay (hs-CRP) with a detection limit of 0.3 mg/L (Immulite analyser, Siemens Healthcare, Erlangen, Germany). TnT was measured by an electrochemiluminescence assay with a detection limit of 3 ng/L (Cobas e411 analyser, Roche Diagnostics, Mannheim, Germany). Nitrite/nitrate (NOx) concentration was determined using a colorimetric non-enzymatic assay (Neogen Corporation, Lexington, KY, USA) following deproteination with zinc sulphate and conversion of nitrate to nitrite using metallic cadmium, nitrite then being measured using the Griess reagent. Samples were analysed in a batch, measurements were performed in duplicate. The limit of detection was 0.5 µmol/L.

Serum levels of IgA- and IgG-class antibodies to Aa and Pg were determined by the multiserotype ELISA as described elsewhere (18). Mixtures of six strains of Aa and three strains of Pg were used as antigens. The two dilutions of samples in duplicate were used: 1:1,500 and 1:3,000 for IgG Aa, and 1:100 and 1:200 for IgA Aa, IgG Pg and IgA Pg. The bound antibodies were visualized using horseradish peroxidase-conjugated goat anti-human IgA or IgG (Sigma) and measured at 492 nm. Unspecific binding was monitored by blank wells, and the results were normalized according to the reference serum samples applied on each plate. The inter assay coefficients of variation were 5.1% for IgA Aa, 5.2% for IgG Aa, 4.4% for IgA Pg and 4.5% for IgG Pg.

-Statistical analysis

Continuous data were summarized as medians and ranges, categorical data as counts and percentages. Patients’ smoking status was classified as currently smoking versus never or previously smoked combined. Univariate statistical analysis was performed for the association of edentulousness with age, sex, body mass index, smoking, hypertension, diabetes, dialysis duration, antibody response and serum levels of CRP, TnT and NOx. The differences between the groups were assessed with the Kruskal-Wallis test for the continuous variables and Fisher’s exact test for the categorical variables. The level of significance was set to α=0.05.

Multivariate linear regression was used for evaluating the independent associations of the covariates on the serum biomarkers CRP, TnT and NOx. Separate models for each of the three biomarkers were constructed, using age, sex, body mass index, smok-ing, hypertension, diabetes, dialysis duration, IgG Aa, IgG Pg, presence of teeth, presence of periodontal pockets and serum levels of the other two biomarkers as potential predictors. The logarithmic transformations of the biomarker levels were needed because of asymmetrical data distributions. Statistical analyses were conducted with the statistical software package R.

## Results

220 patients were invited and 111 agreed to participate in the study. The demographic and clinical characteristics of the participants are shown in  Table 2.

**Table 2:**
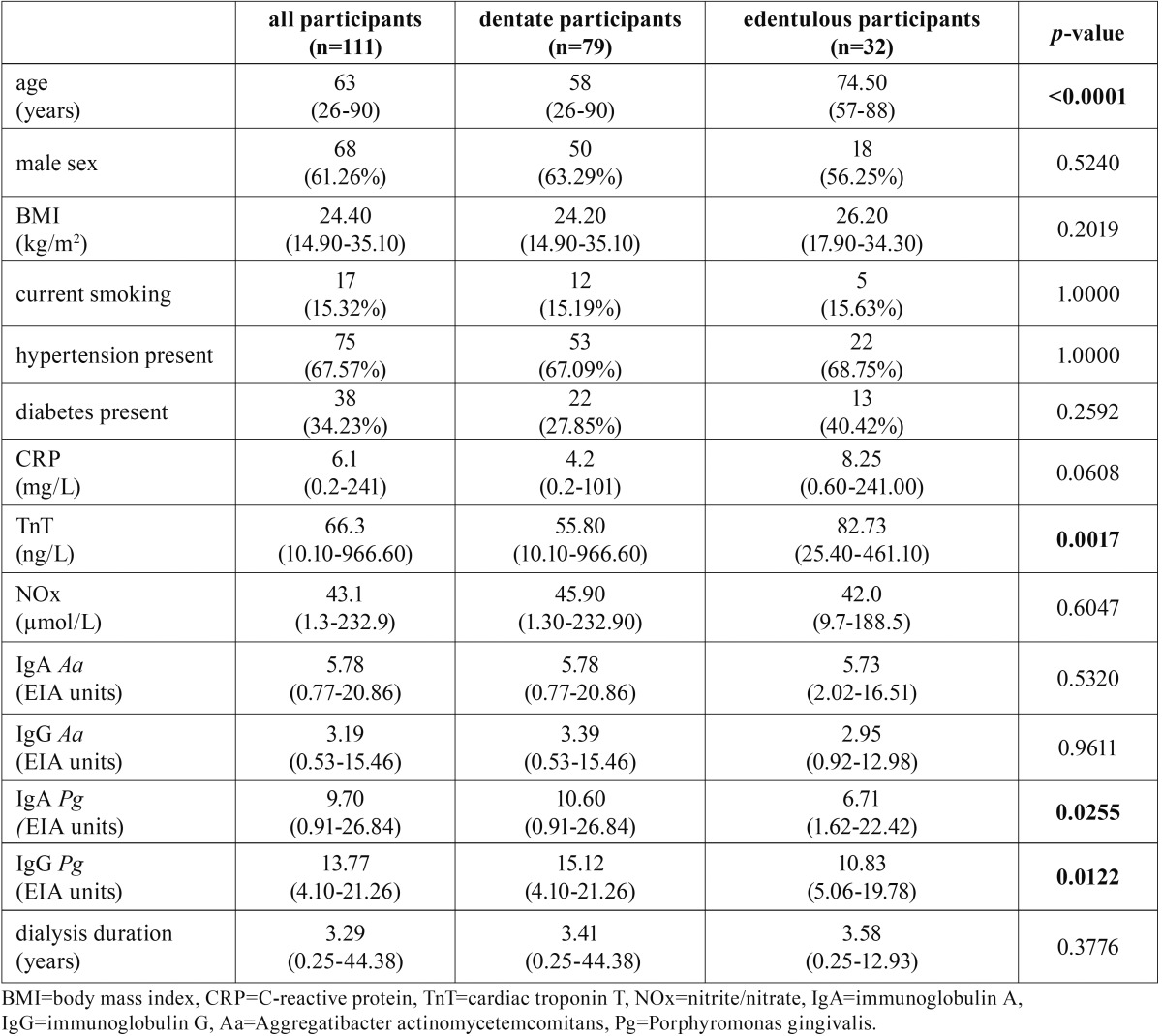
Characteristics of all the participants stratified by the presence of the teeth in the mouth. Medians and ranges are shown for continuous data, counts and percentages (%) are shown for categorical data.

The vascular access for performing the HD was obtained by arteriovenous fistula in 76.6% patients and by venous catheter in 23.4% patients. The most commonly used medicine was calcium carbonate (58.6%) followed by sodium carbonate (42.3%), calcitriol (42.3%), sevelamer (34.2%), furosemide (34.2%), spironolactone (32.4%), acetylsalicylic acid (29.7%), and bisoprolol fumarate (23.4%). Current smoking was reported by 15.3% patients, 31.5% were former smokers and 53.2% had never smoked.

**Figure 1:**
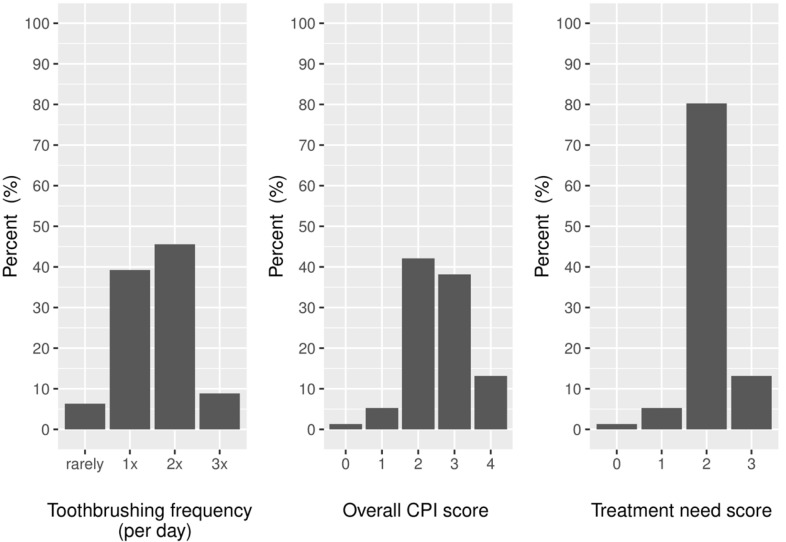
Relative frequencies of toothbrushing habits, overall community periodontal index (CPI) scores and treatment need (TN) scores among the hemodialysis patients.

The typical dentate patient had no decayed teeth, 12 missing and 3 filled teeth, the overall median DMF being 19. The median number of teeth in dentate patients was 13. Visiting their dentist in the last 12 months was reported by 37.8% participants. The majority brushed their teeth once (42.3%) or twice per day (37.8%) (Fig. 1). The median API was 60% (range 12-100%) and the median SBI was 11% (range 0-100%). The CPITN evaluation was performed in 76 out of 79 dentate patients, as 3 patients did not have a sufficient number of remaining teeth. Periodontal health was compromised and the treatment need substantive (Fig. 1). Only one dentate patient had completely healthy periodontal tissues (C0). Bleeding on probing without periodontal pockets (C1) was determined in 5.3% of dentate patients, 42.1% had visible calculus without periodontal pockets (C2), 38.2% had shallow periodontal pockets (C3) and 13.2% had deep periodontal pockets (C4).

**Figure 2:**
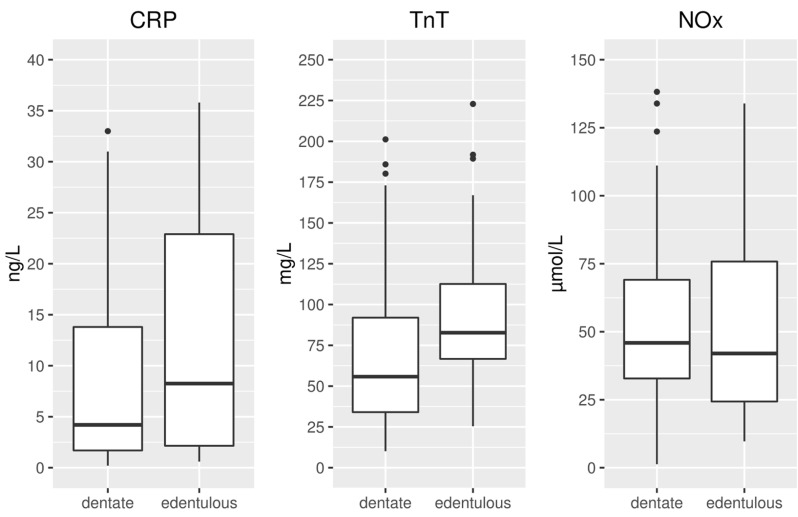
Boxplots of serum biomarker levels in edentulous and dentate hemodialysis patients. To improve the visualization, the plot areas depicting the medians and interquartile ranges are zoomed in and the outermost outliers are not shown. (CRP=high-sensitivity C-reactive protein, TnT=high-sensitivity cardiac troponin T, NOx=nitrite/nitrate).

28.8% participants were edentulous. As shown in  Table 2, edentulism was significantly more prevalent in older patients. Dentate patients had statistically significantly higher levels of antibodies against Pg and also had higher levels of antibodies against Aa, but this difference was not statistically significant. The distributions of serum biomarkers CRP, TnT and NOx in dentate and edentulous patients are shown on Figure 2. The serum level of CRP exceeded the normal threshold of 3 mg/L in 63.1% of the patients. 34.2% exhibited CRP levels higher than 10 mg/L. The serum level of TnT was higher than the normal threshold of 10 ng/L in all of the patients. 25.2% of the patients exhibited TnT levels that exceeded 100 ng/L. The NOx level ranged from 1.3 to 232.9 µmol/L, with the median of 43.1 µmol/L ( Table 2).

Edentulous patients tended to have higher serum levels of CRP and also had significantly higher levels of TnT (Fig. 2 and  Table 2), but the latter association did not remain statistically significant when controlling for age in a multivariate model. Multivariate regression revealed a number of independent predictors for the serum levels of the CRP and TnT ( Table 3). The level of the in-flammatory marker CRP was moderately associated with the variables included in the model (F(13,97)=2.62, *p*=0.0037, adjusted R2=0.16). A positive association with edentulousness (*p*=0.0367) and serum level of TnT (*p*=0.0107) and a negative association with hypertension (*p*=0.0078) was detected. The serum level of the cardiac marker TnT was strongly associated with the variables included in the model (F(13,97)=7.64, *p*<0.0001, adjusted R2=0.44). TnT had a direct positive association with age (*p*<0.0001), hypertension (*p*=0.0159) and the serum level of CRP (*p*=0.0107) and a tendency for a positive association with male sex (*p*=0.0569). Direct negative association of TnT with current smoking status (*p*=0.0043) was detected, as well as a tendency for a negative association with the serum level of NOx (*p*=0.0569), but the overall variability of the biomarker NOx was not explained with the model (F(13,97)=1.47, *p*=0.1443).

**Table 3:**
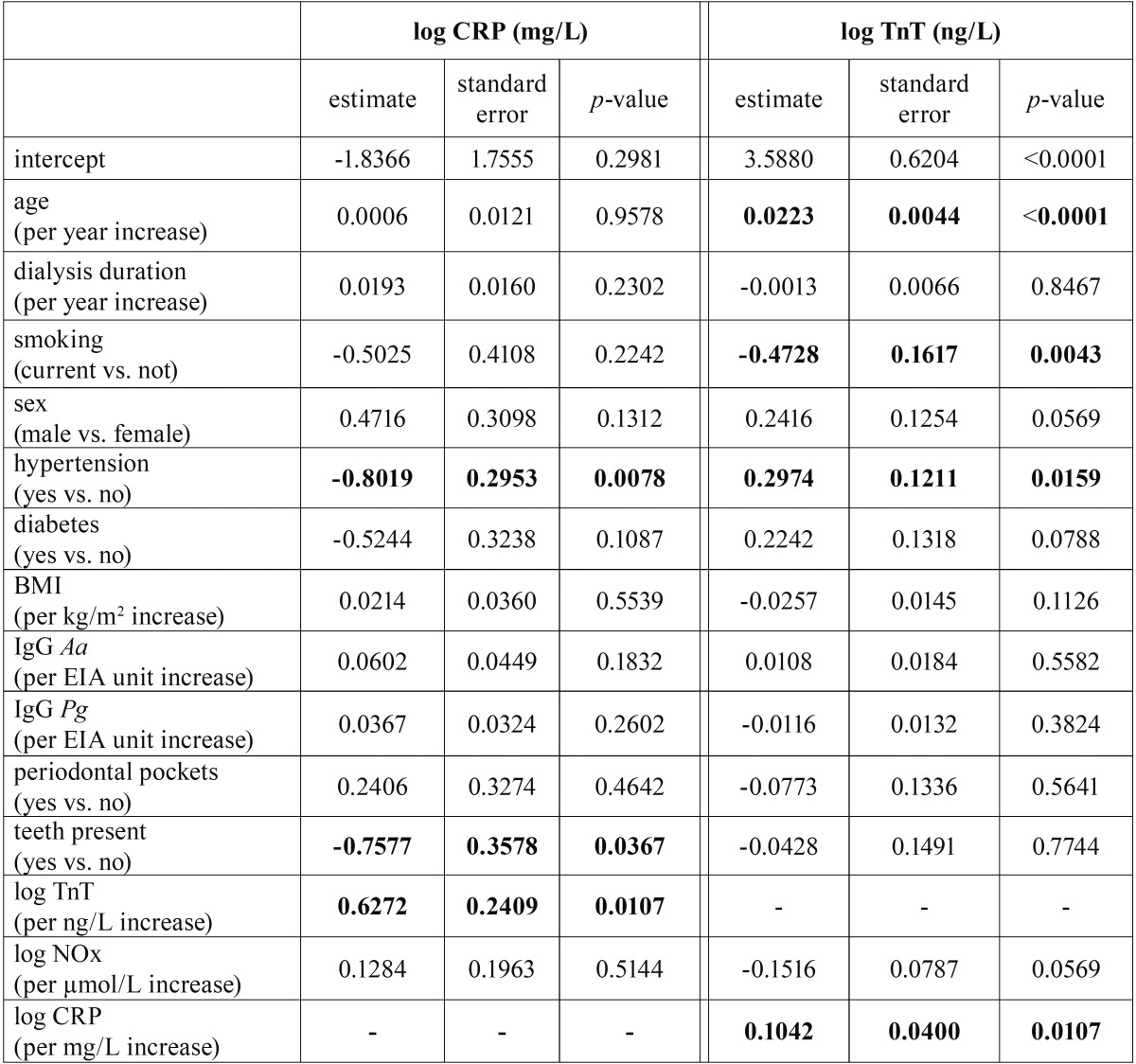
Results of multivariate regression models investigating the independent predictors of C-reactive protein and cardiac troponin T serum levels.

## Discussion

In this cross-sectional observational study, we investigated the oral health of Slovenian HD patients in connection with the serum levels of systemic biomarkers and antibodies against common periodontal pathogens. The oral health of the studied group was generally poor. More than a quarter of the included patients were edentulous and the median DMF in dentate patients was 19. The oral hygiene measures were not sufficient in removing plaque, as evident from the relatively high median API of 60%. The plaque burden was not associated with substantial gingival bleeding upon probing (median value of 11%), but the periodontal health was still a reason for concern. 51.4% of the dentate patients needed scaling and root planing due to periodontal pockets and 13.2% had advanced periodontal destruction requiring surgical periodontal treatment. It was previously demonstrated that the periodontal status among the HD patients is worse compared to healthy controls (19). The present study was originally designed to include a control group of non-HD patients with periodontal disease, which would be matched with the group of HD patients by age and gender. However, out of 200 invited patients only 5% agreed to undergo blood sampling to determine biomarkers. We have therefore proceeded with the HD group only. Alternatively, an interesting comparison may be made to a previous study concerning the oral health of 296 nursing home residents in Slovenia (20), where 56.7% needed scaling and root planing and 21.6% needed periodontal surgery. With the mean age of 80 years, they were older than the current patients on HD (mean age 63 years) and also more likely to be edentulous (35.8% compared to 28.8%), but reported fewer instances of diabetes mellitus (26% compared to 34.2%) and hypertension (47.6% compared to 67.6%) (20).

Due to important interactions between the oral and systemic health, data on serum biomarkers and host response in HD patients was collected in the present study. Although the dentate HD patients had a significantly higher host response against *P. gingivalis*, they were less likely to have increased levels of CRP and TnT compared to edentulous patiens. One of the reasons might be the significantly higher age of the edentulous, as ageing itself contributes to alterations in inflammatory and cardiac biomarkers (21). However, edentulousness as such remained a significant independent predictor of increased CRP even when adjusted for age and other covariates. Tooth loss might impact systemic health via impaired nutrition (22) and mucosal lesions associated with the use of dentures (23), but the underlying relationship is probably more complex. Early loss of teeth is the result of past oral infections and it is likely that these individuals have an inherently increased acute phase response, being more susceptible to various inflammatory processes (8). It was also determined that retention of the teeth, regardless of the presence of periodontal pockets, does not increase CRP beyond the levels observed for edentulous people (8). Dental care and periodontal therapy should therefore aim to retain the teeth in the mouth as long as possible, contributing to prevent malnutrition which is highly prevalent in patients with kidney disease and may also adversely affect the cardiovascular health (24). An authoritative recent review also concluded that tooth loss may be a predictor of shortened longevity (25).

In this study, the overall level of CRP was mildly elevated with the median level of 6.1 mg/L, which is in line with the sub-optimal tissue status in HD patients (7). Values over 10 mg/L, indicative of acute systemic inflammation, were measured in 34.2% of HD patients, compared to 42% in a study by Rahmati *et al.* (26). HD patients with increased levels of CRP were more likely to have higher levels of TnT, confirming a link between the systemic inflammation and a compromised cardiovascular state, even though a direct association of oral health with TnT could not be confirmed in this study. All the participants had detectable TnT levels and three quarters had levels between 10 and 100 ng/L which is considered minimally increased (10). Cardiac biomarkers are commonly elevated in HD patients, but a generally accepted explanation is yet to be found (27). In this study, advanced age and hypertension were the most important predictors of increased TnT. It is interesting that current smokers exhibited significantly lower TnT levels, which is an unexplained phenomenon also reported by others (28). We have also detected a tendency for a negative association of TnT with NOx. This finding corresponds well to the NO vasoregulatory role, but the role of the oxidative stress in both periodontal diseases and ESRD is yet to be determined. The mean NOx level in our study was 54.7 µmol/L (range 1.3-232.9 µmol/L), which is lower than previously reported levels for patients with renal disorders (mean 100.6, range 28.7-216.4 µmol/L), but more dispersed than values for healthy controls from the same study (mean 55.0, range 23.3-110.5 µmol/L) (29). NO has a very complex metabolism, increasing both production and consumption in the general inflammatory, uremic state of the HD patients (15), which might explain the apparently normal mean values measured in this study.

Due to many confounders, it is challenging to investigate the interrelationships of possible risk factors in medically compromised patients. ESRD and periodontal disease are both chronic states associated with systemic inflammation and cardiovascular mortality. The drawback of the observational studies is the lack of causal inference, but intervention trials testing the hypothesis that treatment of periodontal diseases improves the chronic kidney disease-related morbidity, are yet to be performed (30). In light of evidence that periodontal treatment decreases CRP and other biomarkers of cardiovascular disease (31), the prophylaxis, early dental care and periodontal maintenance should be intensified in ESRD patients as this may have a beneficial impact on their general, and especially cardiovascular health.

## Conclusions

This study provides evidence of compromised oral health status and increased systemic levels of inflammatory and cardiac bio-markers of HD patients in Slovenia, with edentulousness as an independent predictor for the increased CRP.
